# Supplementary material for: Are carbon emissions trading and green financial instruments synergistic? -Comprehensive quantitative research based on content analysis
Source: PLoS One. 2024 Mar 7;19(3):e0298601. doi: 10.1371/journal.pone.0298601 (PMC10919601; doi:10.1371/journal.pone.0298601)
Supplement: S1 Data — (ZIP) [file pone.0298601.s001.zip › Data/地级市Z_F工作报告.zip!/╔╜╢1⁄2/═■║ú╩╨/└┤╫╘╣1⁄2╓┌║┼í╛┬φ┐╦╩2╛▌═°í┐_2006.docx]

各位代表：

现在，我代表市人民政府，向大会作政府工作报告，请予审议，并请市政协委员和其他列席人员提出意见。

一、“十五”时期政府工作的回顾

过去的五年，是我市发展进程中不平凡的五年。五年来，在省委、省政府和市委的正确领导下，市政府团结和带领全市人民，高举邓小平理论和“三个代表”重要思想伟大旗帜，深入贯彻党的十六大和十六届三中、四中、五中全会精神，坚持以科学发展观统领经济社会发展全局，全面落实市委“一一六一”发展目标和思路，认真执行市人大及其常委会的各项决议，全市改革开放和现代化建设取得可喜成就，经济社会进入新的发展阶段。

（一）综合实力跃上新台阶。五年来，我们始终牢牢抓住发展这个第一要务，以解放思想为先导，以富民强市为目标，坚持辩证思维，把落实国家宏观调控政策作为又快又好发展的重大机遇，适时调整发展思路，着力解决影响经济社会发展的突出问题，不断引导经济社会持续快速健康发展。去年全市生产总值达到1169.8亿元，年均增长15.8%，增幅比“九五”时期提高2.5个百分点；人均生产总值达到5827美元，继续在全省保持领先地位；财政总收入超过110亿元，其中地方财政收入57.5亿元，年均分别增长29.8%和33.1%。在中国社科院发布的城市综合竞争力排名中，我市由第42位提升到27位；所辖三市全部进入全国百强县行列，并实现了位次前移。

（二）结构调整取得新成效。五年来，我们坚持“抓二产带一产促三产”的思路，扎实推进“三大基地”建设。三次产业比重调整为9.2：62:28.8。围绕制造业基地建设，培植了一批支柱产业、骨干企业和知名品牌。去年全市规模以上工业实现增加值672亿元，年均增长22.1%，其中五大产业群的比重达到81.1%，销售收入过50亿元的企业达到3家，高新技术产业产值占工业总产值的比重由12.9%提高到24.9%。中国名牌产品从无到有，总数达到8个；中国驰名商标由2个增加到7个，总量分别跃居全省第五和第二位。围绕旅游度假基地建设，推进了旅游资源的整合与深度开发，繁荣了晚间市场和休闲经济，创建了全国第一个“中国优秀旅游城市”群。去年全市实现旅游总收入85.5亿元，年均增长17.5%；社会消费品零售总额达到284.8亿元，年均增长13.7%。围绕以海产品为主的农副产品出口加工基地建设，深入推进了农业产业化、标准化和国际化进程，新增国家级标准化示范区7个，年粮食总产稳定在95万吨左右，较大规模的畜牧养殖场由230家发展到681家，名优水产养殖比重由35%提高到64%，远洋渔船由28艘增加到185艘。

（三）对外开放再创新局面。五年来，我们抢抓国际资本转移和国内南资北上的有利机遇，坚持对内对外开放一齐抓，在更高层次上打好“开放牌”，举办了东北亚经济合作论坛、东亚投资论坛等一系列重大涉外活动，组织了赴韩国、日本、香港、欧美及上海、温州、西安、哈尔滨、东莞等一系列国内外招商促进活动，全方位对内对外开放战略实现了新突破。五年累计实际利用外资42.6亿美元，是“九五”期间的4倍，年均增长41.9%。实际利用内资580亿元，是“九五”期间的5倍多，年均增长48%。投资过千万美元的外资项目、过亿元的内资项目分别达到320个和136个。去年进出口总值达到75.5亿美元，其中出口47.3亿美元，分别是2000年的3.8和3.6倍，年均增长30.4%和29.3%。五年内完成对外工程承包营业额4.5亿美元，外派劳务2.1万人次，分别是“九五”时期的2.3倍和1.7倍。按要求清理整顿了各类开发区和园区，两个国家级开发区、出口加工区及省级开发区、旅游度假区的发展步伐进一步加快。

（四）体制改革实现新突破。五年来，我们坚持市场化取向，积极推进各项改革，不断增强发展活力。企业改革向纵深推进，基本完成县以上企业改制任务，镇村企业改制面达到80.1%，比2000年提高65个百分点。组建了国资委和国资公司，理顺了国有资产监管体制。企业上市取得重点突破，先后有5家公司在境内外上市。民营经济加速发展，非公有制经济占生产总值的比重由36.8%提高到57%。财税体制改革不断深化，在全省率先推行了公务通讯补贴和公务用车货币化改革，推进了政府采购和国库集中支付改革。农村税费改革扎实推进，在全省率先取消了农业税，并停征了乡村公益事业金，实行了村干部工资财政补贴制度，落实了粮食直补、良种补贴、农机补贴等政策，切实减轻了农民负担。加强了与国家开发银行的合作，建立了融资新机制。推进了农村信用社改革，加大了金融安全区的创建力度，改善了金融生态环境。理顺了食品、药品市场监管机制，整顿规范了市场经济秩序。深入推进了事业单位改革，压缩事业编制1万多个。深化了户籍制度改革，建立了城乡统一的户籍登记管理制度。基本完成了粮食购销企业改革，投资、价格、医疗卫生等改革也取得新的进展。

（五）城乡面貌发生新变化。五年来，我们坚持在更高层次上打好“环境牌”，构筑更加和谐的生态和人居环境。累计完成固定资产投资1866亿元，是“九五”期间的4.7倍，年均增长48.9%。高标准实施了环海公路、桃威铁路改建、威海港三期、威海机场改造等交通重点工程，新建改建公路710公里；新增海上航线19条，港口吞吐量由762万吨提高到2219万吨；开通了至汉城（仁川）国际空中航线，机场旅客吞吐量由6万人次提高到21.3万人次。中心城区完成了世昌大道、环山路、青岛路、机场路等城市干道工程，建成了海滨、海港等11个开放式公园及一批社区花园，新建了国际展览中心、体育场等公益设施，新增日供水能力7万吨、日污水处理能力4万吨，建成区面积由43.7平方公里扩大到82平方公里。荣成、文登、乳山也相继完成了一批城市基础设施重点工程，城市的支撑和保障能力进一步增强。围绕建设一体化威海，完成了新一轮城市总体规划修编，确立了新的城市定位，加快了市区对接步伐。建制镇由72个调到50个，城市化率由42%提高到53%。倡导和树立了“生态是借贷而非继承”的理念，出台了《威海生态市总体规划》，实施了“青山、碧海、蓝天、宁静”行动，开展了城乡环境综合整治，狠抓了造林绿化工作。全市森林覆盖率达到36.8%，提高了8个百分点。先后成为全国第一个“国家环境保护模范城市”群、全省第一个“园林城市”群，荣获了联合国“人居奖”，成功举办了两届中国威海国际人居节，最适合人类居住和创业的城市品牌越来越响亮。

（六）人民生活水平有了新提高。五年来，我们始终把人民群众的利益放在首位，坚持每年办一批实事、好事，着力解决群众在就业、生活、就医、住房、子女上学等方面的实际问题。加强了农村“路水电医学”建设，基本实现了村村通油路，全面完成了农村电网改造，农村自来水普及率达到58.8%，村容村貌和农村精神文化生活都有所改善。投入8.8亿元，新建改建中小学校舍130万平方米，缓解了城区中小学班额过大的问题。开发经济适用房32.9万平方米，为3956户低收入居民解决了住房问题。强化了就业和社会保障工作，新增城镇就业人口22.5万人，转移农村劳动力9.4万人，城镇登记失业率一直控制在1.7%以内，城镇职工养老、医疗等保险覆盖面均保持在98%以上。建立了失地农民基本生活保障制度、建筑行业农民工工资保障金制度，出台了城乡最低生活保障对象医疗教育住房救助办法，对5.2万名低保对象实施了保障。对受灾群众、城乡大病致贫家庭和困难大学生进行了及时救助。改扩建敬老院49处，对9134名五保对象实行了应保尽保。去年城镇居民人均可支配收入达到12455元，农民人均纯收入达到6083元，年均分别增长10.7%和9.6%。

（七）和谐威海建设谱写新篇章。五年来，在经济又快又好发展的同时，科教文卫体等社会各项事业也得到了全面发展。我们坚持打好“科技和人才牌”，组织实施市级以上科技计划1000多项，取得重要科技成果265项；建设国家863计划成果产业化基地5个，新增市级以上企业技术开发中心和工程技术研究中心97家，总数达到115家；与驻威高校共建了哈工大国际微电子技术中心、山大国际生物技术研发中心和宋健科学技术研究院；设立了13个博士后科研工作站和工作企业，引进各类人才2.2万人，被评为“全国科技进步先进市”，并被确定为“全国信息化试点城市”和“国家知识产权试点城市”。巩固和完善了“以县为主”的义务教育管理体制，调整优化了中小学校布局，教育工作综合发展水平名列全省第一，普通高考本科上线率和人口万人比连续12年居全省首位；推进了校地共建，高校全日制在校生由0.6万人增加到2.9万人；整合了职业技术教育资源，搬迁扩建了威海职业（技术）学院、威海工程技术学院，引进了山东药品食品职业学院、山东外事翻译学院，提高了技能型人才的培养能力。建立和完善了公共卫生体系，新建了市级传染病医院、市疾控中心国家标准实验室，新型农村合作医疗制度覆盖面达到90.7%，城乡预防、保健、医疗网络进一步健全。出色完成了“非典”防控工作，禽流感防治也取得了阶段性成果，确保了一方净土。人口和计划生育工作成效显著，实行了农村和城镇失业（无业）人员独生子女父母奖励费财政统一发放制度，开展了农村部分计划生育家庭奖励扶助制度试点，继续保持了低生育水平。文化体育事业蓬勃发展，推出了海权、梦海等特色文化品牌，向公众免费开放了科技文化体育设施，丰富了群众性文化活动；举办了23次省级以上体育赛事，全民健身活动更加普及。对口支援和重点帮扶工作取得了阶段性成效。广播电视、新闻出版等事业繁荣活跃，文物保护得到加强。国防动员、人民防空、民兵预备役和“双拥”共建工作迈出新步伐。外事、侨务、对台工作取得新成绩。妇女儿童、老年人、残疾人权益得到有效保障。民族、宗教、史志、档案、气象、地震等事业都有了新发展。开通了威海信用网，完善了社会信用体系，“诚信威海”建设取得实效。“平安威海”建设深入推进，刑事案件、治安案件和各类安全事故明显下降，三市三区均达到省“平安县市区”标准。弘扬了“自强不息、创新创业、海纳百川、追求卓越”的威海精神，民主法制、精神文明建设成效显著。

（八）政府自身建设出现新气象。五年来，我们按照为民、务实、清廉的要求，扎实推进了法治政府、阳光政府和责任政府建设。各级政府深入开展了保持共产党员先进性教育活动和思想解放大讨论活动，着力解决了影响政府效能和作风建设的实际问题。认真贯彻实施了《行政许可法》，市县两级都成立了行政审批中心，完成了两轮行政审批制度改革，开展了相对集中行政处罚权工作。坚持定期向人大和政协通报政府工作，认真办理人大代表建议和政协委员提案，邀请部分人大代表、政协委员及普通市民列席市政府重要会议。大力推行政务公开，加强了电子政府建设，建立了“中国威海”政府门户网站，开通了“12345”市长公开电话和市长电子信箱，开办了行风热线和直播威海栏目，及时解决群众生产生活中遇到的困难和问题，行风热线的做法还受到党和国家领导人的充分肯定，并得到了主流媒体的广泛宣传。强化了“三个体系”建设，营造了干事创业的浓厚氛围。加强了政府机关廉政建设，严肃查处了一批违法违纪案件，政风行风都有新的转变。

各位代表，刚刚过去的一年，全市上下认真贯彻落实胡锦涛总书记视察威海时的讲话精神，团结一心，迎难而上，圆满完成了市十四届人大三次会议确定的各项任务。全市生产总值比上年增长17.4%，地方财政收入增长35%，固定资产投资增长25.1%，社会消费品零售总额增长15.7%，实际利用外资增长22.8%，实际利用内资增长48.6%，外贸出口增长37.2%，城镇居民人均可支配收入增长12.1%，农民人均纯收入增长13.2%。在经济社会快速发展的同时，我们还集中力量办成了一批事关威海发展的大事，威海机场正式对外开放，威乳高速公路全线开工，荣乌高速公路荣文段、青烟威城际铁路项目已列入省和国家计划；石岛管理区正式成立，威海工业园的建设全面启动，并取得实质性进展。去年我市还被评为“2005中国十佳宜居城市”之首、“2005内地城市投资环境排行榜”第20位、欧洲游客最喜爱的十大中国旅游城市之一，荣获了“全国节水型城市”、“全国社会治安综合治理优秀地市”、“创建全国文明城市工作先进城市”等称号。这些，都使我们的比较优势更加突出，基础设施更加完善，城市品牌更加响亮，从而为“十一五”的发展奠定了更加坚实的基础。

各位代表！去年我们按照国务院和省政府的要求，制定颁布了《突发公共事件总体应急预案》和35个专项预案，经受了强热带风暴、禽流感防治，尤其是年末暴风雪的严峻考验。面对这场200年未遇的暴风雪的突然袭击，全市上下在市委、市政府的坚强领导下，广泛动员，积极应对，万众一心，打赢了一场抗击暴风雪的人民战争，真正做到了有大的灾情，无大的灾害，引起了国际国内的广泛关注，各大媒体进行了深入广泛的报道，受到了中央和省领导的表扬，进一步树立了威海的良好形象。

各位代表！五年的辉煌令人振奋，五年的成就来之不易。成绩属于党、属于人民、属于为威海建设付出心血和汗水的各界朋友。在这里，我代表市人民政府，向给予我们支持与监督的人大代表、政协委员、各民主党派、工商联和无党派人士、各人民团体、社会各界人士，向各位老领导、老同志，向驻威部队指战员、武警官兵和政法干警，向所有关心支持威海建设的港澳台同胞、海外侨胞和国际友人，向所有的纳税人，致以崇高的敬意和衷心的感谢！

各位代表！回顾五年来的工作，我们深切体会到，要不断开创改革开放和现代化建设的新局面，必须根据形势的要求和发展的需要，不断解放思想、更新观念、深化改革，消除发展中的各种思想和体制障碍；必须坚持辩证思维，做好结合的文章，在与时俱进、抢抓机遇中创造性地开展工作；必须认清自身的优势和劣势，及时调整发展思路，千方百计加快发展；必须以科学发展观统领全局，集中力量解决突出矛盾和问题，推进经济社会统筹协调发展；必须坚持以人为本，让人民群众在改革和发展中得到更多实惠，最大限度地调动全市人民的积极性和创造性。这些经验，凝聚了全市上下的集体智慧，是我们未来发展的宝贵财富。

在总结成绩的同时，我们也要清醒地看到，我市经济社会发展中还存在一些突出的问题。主要是：经济的结构性矛盾还比较突出，服务业发展还相对滞后，财税“三个比重”仍然偏低，投资和消费关系尚待优化；粗放型经济增长方式没有根本转变，自主创新能力还不够强；涉及群众切身利益的不少问题亟待解决，农民增收的长效机制还有待完善，收入分配中的矛盾还比较突出；生态还比较脆弱，资源利用效率有待提高；制约经济社会发展的体制机制问题还比较多；少数部门依法行政的能力和水平有待进一步提高，工作作风、行政效能有待进一步改善，腐败现象和不正之风还时有发生，等等。对此，我们一定要有针对性地采取措施，在今年和“十一五”的工作中认真加以解决。

二、“十一五”时期的指导思想和目标任务

各位代表！从今年开始，我们已进入第十一个五年规划时期。根据国家、省的部署和市委的要求，市政府编制了《威海市国民经济和社会发展第十一个五年规划纲要（草案）》。《纲要（草案）》及《说明》已印发大会，请一并予以审议。

“十一五”时期是我市实现“翻三番”和“两个率先”目标的关键时期。根据科学发展观的要求，综合考虑国际、国内形势和威海的实际情况，确定“十一五”时期我市经济社会发展的指导思想是：坚持以邓小平理论和“三个代表”重要思想为指导，以科学发展观统领经济社会发展全局，全面落实市委“一一六一”发展目标和思路，以改革开放为动力，以自主创新为支撑，以提高经济运行质量和效益为目标，突出抓好经济结构调整和经济增长方式的转变，大力建设现代制造业基地、旅游度假基地和以海产品为主的农副产品出口加工基地，全面建设活力、平安、文明、民本、生态、精品威海，推进经济社会又快又好发展，为建设全面小康和现代化的和谐威海奠定坚实基础。

综合考虑发展阶段、发展环境和基础条件，充分体现科学发展观、构建和谐社会和全面建设小康社会这三大时代主题，《纲要（草案）》围绕综合实力、经济结构、开放型经济、城市化、循环经济、人民生活等方面，提出了“十一五”期间的奋斗目标和任务。

（一）着力推进又快又好发展，综合实力再上新台阶。继续坚持“抓二带一促三”的思路，更加注重经济增长方式的转变，更加注重资源节约和生态保护，大力实施工业强市、三产兴市战略，加快建设“三大基地”，构建更具国际竞争力的现代产业体系，构建资源节约型和环境友好型社会，推动经济社会发展切实转入科学发展的轨道。到2010年，全市生产总值提前实现第二个翻番，突破2000亿元，年均增长13%；三次产业结构调整为8：60：32；地方财政收入年均增长15%；社会消费品零售总额年均增长13%；森林覆盖率达到40%；万元生产总值能耗降低20%以上。

（二）着力实施“科教兴威”和“人才强市”战略，自主创新能力显著提高。坚持把提高自主创新能力作为科技发展的战略基点和优化产业结构、转变增长方式的中心环节，加大政府引导力度和科技创新投入强度，逐年提高科技开发投入占生产总值的比重，加快发展高新技术产业，提高原始创新、集成创新和引进消化吸收再创新的能力。进一步巩固教育的基础地位，加强人才队伍建设，全面提升人力资源素质，不断增强区域竞争力。到2010年，高新技术产业产值占工业总产值的比重达到30%。

（三）着力建设社会主义新农村，城乡发展更加协调。坚持“多予少取放活”的方针，加大工业反哺农业、城市支持农村的力度，全面推进社会主义新农村建设，构建以城带乡、城乡联动的区域发展格局。坚持以打造一体化威海、建设国际精品城市为目标，加快市区对接，进一步优化城市形态和功能布局，加快构建“A”字型城市发展框架。到2010年，全市城市化率达到60%，中心城市人口增加到100万人，建成区面积扩大到125平方公里。

（四）着力推进改革开放，发展活力明显增强。坚持以市场化改革为方向，以体制创新和制度建设为重点，全面推进经济、社会、政府管理各领域的改革，并要在一些关键领域和重要环节上取得突破。进一步树立全球视野，更加积极有效地利用两个市场、两种资源，妥善应对开放风险和挑战，提高对内对外全方位开放的层次和水平。“十一五”期间，外贸出口年均增长15%，累计利用外商直接投资50亿美元、实际利用内资1000亿元。

（五）着力加强基础设施建设，支撑保障能力进一步提升。“十一五”期间，围绕“公路升级、铁路提速、港口扩建、机场开放”，开工并完成荣乌高速公路荣文段、青烟威城际铁路和威海港三四期扩建工程等一批重大交通设施项目，做好中韩铁路轮渡项目的论证和争取工作，搞好铁路、水路、航空的相互衔接和配套。到2010年，国省道干线公路通车里程达到1200公里，港口吞吐量力争达到1亿吨。以发展新能源为重点，开工建设一批风能、水能、核能等重大电力项目。搞好污水处理厂的改造与建设，力争到2010年城市污水集中处理率达到80%以上。积极搞好水利、信息基础设施等建设，形成适度超前、持续发展、保障有力的城乡基础设施体系。五年累计完成固定资产投资3000亿元。

（六）着力促进人的全面发展，和谐威海建设取得更大进步。坚持以人为本的指导思想，把强市富民、改善民生放在更加突出的位置，认真解决城乡群众最关心、最迫切的问题。在注重效率的同时，兼顾各方面利益，更加注重社会公平，促进城乡共同发展、社会共同进步、群众共同富裕。“十一五”期间，城镇居民人均可支配收入年均增长8%，农民人均纯收入年均增长8%，人均期望寿命达到77岁，人口自然增长率保持稳定，城镇登记失业率控制在3%以内。

在此基础上，再经过十年的努力，到2020年，全市生产总值再翻一番，突破4000亿元，人均生产总值达到1.5万美元，提前基本实现现代化。

三、2006年政府工作安排

2006年是实施“十一五”规划的开局之年。做好今年工作，开好局、起好步，必须按照市十二届四次党代会的要求，坚持富民优先、科教优先、环保优先、节约优先，努力实现又快又好发展。主要预期目标为：全市生产总值增长14%，地方财政收入增长20%，万元生产总值能耗降低4%，固定资产投资增长20%，社会消费品零售总额增长14%，外贸出口增长20%，外商直接投资增长10%，实际利用内资增长20%，城镇居民人均可支配收入增长10%，农民人均纯收入增长8%，城镇登记失业率控制在2%以内，人口自然增长率控制在0.6‰以内。要完成以上预期目标，必须突出抓好以下九项重点工作：

（一）扎实推进社会主义新农村建设。按照生产发展、生活宽裕、乡风文明、村容整洁、管理民主的要求，坚持规划先行，有步骤、有计划、有重点地扎实推进，确保今年在社会主义新农村建设上迈出有力的一步。

一是大力发展富有威海特色的现代农业。以增加农民收入为目标，紧紧围绕以海产品为主的农副产品出口加工基地建设，加快农业和农村经济结构调整步伐。在确保粮食安全的前提下，积极发展林果、蔬菜、中药材、花生四大主导作物，粮经比例调整到44：56。扩大奶畜及特种动物养殖规模，全年奶类产量增长12%。加快发展以远洋渔业、名优养殖、精深加工为重点的现代渔业，名优海产品养殖比重提高到65%，水产品精深加工比重达到70%。加强农田基本建设，年内改造中低产田5万亩，改善恢复扩大灌溉面积16万亩。大力扶持农业龙头企业，积极培育农产品名牌，加快农业标准化、产业化和国际化进程，力争新增省级重点龙头企业6家以上。

二是扩大公共财政对农村的覆盖面。全面落实已出台的各项惠农政策，今年全市安排农村方面的财政支出13.2亿元，增长19.8%，其中市级1.1亿元，增长32.8%。主要是：（1）安排6.9亿元，进一步加强农村教育。（2）安排5100万元，改善农村医疗条件，落实计划生育奖励政策。（3）安排3700万元，落实粮食直补、农机补贴等惠农政策，扶持农村经济合作组织。（4）安排8000万元，推进荒山和道路绿化。（5）安排900万元，落实奶业扶持政策，抓好禽流感等动物疫病防治。（6）安排3800万元，解决和改善450个村的饮水条件，村村通自来水普及率达到78%。（7）安排2500万元，提高农村的五保和最低生活保障水平。（8）安排2400万元，落实村干部工资财政补贴政策。（9）安排500万元的社会主义新农村建设专项资金。同时，鼓励社会资金投向农业和农村，建立健全良性投入机制。

三是全面深化农村综合改革。巩固农村税费改革成果，稳定粮食价格，加快乡镇机构、农村义务教育和县乡财政体制改革，防止农民负担反弹。深化土地征用制度改革，切实保障农民的土地权益。从今年起全面取消农民工和城镇职工的界限，加强对农村劳动力转移的组织引导、信息服务和职业技能培训，健全维护进城务工人员权益的保障机制，全年转移农村劳动力2万人。

四是稳步推进镇村规划建设。坚持以城带乡，积极推动城镇规划和基础设施向农村延伸，力争今年完成全市新农村建设的总体规划和重点镇、示范村建设规划。深入实施“百村示范、千村整治”工程，广泛开展农村环境综合整治，各市区都要抓1－2个示范镇（办事处），各镇（办事处）要抓3－5个示范村，通过示范效应，带动全市镇村环境有新的改变。

五是加强农村文化建设。加快县级文化馆、图书馆和镇文化站、村文化室等农村公共文化设施建设。深入开展“三下乡”活动，普及科学知识和法律知识，丰富农村精神文化生活，不断提高农民的思想文化素质和农村文明程度，培养社会主义新型农民。

（二）加快现代制造业基地建设。坚持走新型工业化道路，加速工业结构优化升级，做大做强现代制造业。

一是大力发展产业集群。按照“大项目、产业链、产业群、制造业基地”的发展思路，加快用高新技术和先进适用技术改造提升传统产业，进一步拉长产业链条，培植壮大五大产业群。以电子信息、生物技术、新材料和海洋技术四大领域为重点，集中力量抓好碳纤维原丝等11个高新技术产业项目建设。到年底，力争五大产业群增加值占生产总值的比重提高2个百分点，高新技术产业产值占工业总产值的比重提高2个百分点。

二是做大做强骨干企业。认真落实市里已出台的各项扶持政策，以60户市级重点企业和40户成长性好的中小企业为载体，抓好110个投资过亿元的重点项目建设，做大做强现有企业，力争年内销售收入过50亿元的企业达到4家以上。

三是积极实施品牌战略。全面落实品牌战略实施意见和奖励办法，引导和支持优势企业加强品牌宣传，开展品牌经营，放大品牌效应，真正走上“以人才兴科技、以科技上质量、以质量拓市场、以市场树品牌”的发展之路。年内力争新增1－2个中国名牌产品和中国驰名商标。

（三）下大力气推动服务业发展。服务业是我市经济发展的薄弱环节，必须作为今后投资的重点、工作的着力点和发展的增长点。

一是加快发展旅游业。以福文化为主题，以千公里海岸为依托，全面实施“幸福海岸专项规划”，深度开发和整合旅游资源，抓好刘公岛空中快车及渔人码头、福如东海、乳山口福地养生园、黄金主题公园及温泉度假村等10个重点旅游项目建设。大力开拓国内外旅游市场，开发一批旅游新产品，推动观光型旅游向休闲度假型旅游转变。年内旅游总收入达到96亿元以上。

二是积极发展现代服务业。以市场化为导向，加强与国内外会展组织的合作，加快国际会议中心建设，高水平办好第三届国际人居节，推动会展业与旅游、商贸、文化、体育产业融合发展。大力推广新型物流业态，培植一批大型流通企业集团、物流中心和配送中心。银行、保险、证券业等是现代服务业的重要领域，又是我市服务业中的薄弱环节，必须提高重视程度，加大扶持力度，加快发展速度，年内要研究出台具体的鼓励政策和措施，推动银行、保险、证券等产业的快速健康发展。金融生态环境是当前发展软环境的一个重要标志，要作为改善发展环境的重要方面来抓，认真落实金融生态环境建设目标责任制，加快建立和完善信用信息基础数据库，坚决打击恶意逃废债务的行为，完善社会诚信体系。继续实施好“百项重点银企互动工程”，促进银行和企业双赢发展。同时，要积极创造条件，加快发展信息咨询、社区服务、中介服务等现代服务业，不断提高服务业发展层次和水平。

三是努力扩大消费需求。坚持把扩大消费需求作为拉动经济增长的基本立足点，不断提高城乡居民特别是农民和城镇低收入居民的收入水平，稳定消费预期，扩大即期消费。搞好城市中心商业区建设，优化商业网点布局，努力做活早市，做旺午市，繁荣夜市。支持连锁超市向农村延伸，加快农村现代流通服务体系建设，进一步改善农村消费环境。落实住房、汽车等消费政策，扩大文化、娱乐、健身等服务性消费。加强粮食市场监管。大力整顿和规范市场秩序，严厉打击制售假冒伪劣商品行为，切实保护人民群众消费安全。年内，力争销售收入过亿元的商贸流通企业发展到15家。

（四）不断增强自主创新能力。科技、教育、人才是构建区域核心竞争力和增强持续创新力的关键，必须坚持不懈地打好“科技和人才牌”，加快建设创新型社会。

一是建立健全科技创新体系。坚持政府支持、企业主导、产学研相结合，继续抓好威海国际生物技术研发中心、威海国际微电子技术中心、宋健科学技术研究院以及国家863成果转化基地、科技孵化器、博士后科研工作站、企业技术中心等创新载体建设。研究制定支持企业科技创新的政策措施，今年用于中小企业科技创新的财政资金2000万元，拨付科技三项经费1700万元，组织实施市级以上科技计划180项。要研究完善这些财政资金的投入机制，切实起到鼓励创新和“四两拨千斤”的作用，支持企业和科研机构对关键、共性和配套技术进行联合攻关，攻克一批核心技术，开发一批具有自主知识产权和市场竞争力的产品，推动“威海制造”向“威海创造”转变。要加强科技中介服务体系建设，完善科技创新支撑体系。年内市级以上技术开发中心和工程研究中心分别增加10家以上。

二是优先发展教育事业。不断改善办学条件，巩固提高义务教育质量，市区重点抓好威海四中和二职专扩建、城里中学搬迁改造等工程，各市区也要根据需要搞好中小学校的扩建改建。认真落实“两免一补”政策，严格规范教育收费行为，加大对农村教育的投入，今年市县两级安排财政资金3200万元，从2006年新学年开始，对农村义务教育阶段学生全部免收学杂费。大力发展职业技术教育，在继续办好威海职业（技术）学院和威海工程技术学院的基础上，整合发展县域职业技术教育资源，搞好成人教育和继续教育，多渠道培养应用型、技能型人才。积极探索校地共建的良性机制，促进驻威高校快速发展，不断提高合作办学水平。

三是深入实施“人才强市”战略。牢固树立人才是第一资源的观念，全面加强人才资源能力建设。制订和实施相关政策，引进和培养一批掌握核心技术、拥有发明成果的拔尖人才，引进和培养一批善于组织科技研发团队、能够统筹科技资源与生产要素配置的领军人才，同时要善于“借鸡下蛋”，借用外地人才为我所用，帮助我们提高创新能力。

（五）切实提高开放的层次和水平。深入实施互利共赢的开放战略，广泛参与国内外经济技术交流与合作，在更大范围、更广领域、更高层次上构建全方位开放格局。

一是着力提高利用内外资的质量。抓住国际产业转移和国内南资北上的有利机遇，按照“文化搭台、旅游推介、经贸唱戏”的思路，继续突出对韩招商，加大对港台、东南亚和欧美的招商力度，扩大与国内长三角、珠三角、东北及京津塘等重点地区的合作，建立起重点带动、多元并进的招商格局。抓好产业和基础设施招商，力争在“三大基地”建设尤其是造船、食品加工、旅游等方面有较大突破。突出大项目招商，积极吸引跨国公司和国内大企业集团在威海设立研发中心和制造基地。全年实际利用内资达到276亿元，外商直接投资达到14亿美元以上。

二是高标准搞好园区建设。坚持一次规划、分步实施的原则，加大投资力度，切实提高园区的规划建设水平。抓好威海工业园建设，引导重点内外资项目和骨干企业向园区集中，打造现代制造业聚集的新型工业区。深入实施“以港兴市”战略，加快推进临港工业园建设。两个国家级开发区和出口加工区要充分发挥示范、辐射和带动作用，在扩大规模的同时，不断提高发展的质量和水平。各个省级开发区要按照集约开发、突出特色、错位竞争的原则，搞好载体建设，不断增强承载力和竞争力。

三是大力开拓国内外市场。牢固树立国内外统一大市场的理念，引导企业强化营销队伍和销售网络建设，不断扩大我市产品的国内外市场占有率。深入实施“科技兴贸”战略，扩大机电产品和高新技术产品出口，实现由数量创汇型向质量效益型转变。加快加工贸易转型升级，引导现有加工企业把高技术含量、高附加值的生产环节转移到我市。积极实施“走出去”战略，支持有条件的企业开发国内国外资源、参与国际国内承包工程投标，扩大劳务输出。今年进出口总额要突破90亿美元，外派劳务超过5000人。

（六）积极深化和完善市场化取向改革。坚持以建立现代企业制度为目标，加快县以上困难企业及镇村企业改制步伐，搞好已改制企业的规范运作，年内镇村企业改制面达到85%以上。大力培植上市资源，支持有条件的企业在国内外证券市场上市和再融资。健全国有资产管理体制和监督方式，建立国有资本经营预算制度，确保国有资产保值增值。加大事业单位改革步伐，稳妥推进市政公用事业改企改制工作。

加快推进公共财政体系建设，强化社会综合治税，完善税收征管体制，确保“三个比重”有新的提高。继续深化国库集中支付改革，强化预算约束，优化支出结构，提高财政资金使用的规范性、安全性和有效性。加强对政府投资的管理，实行投资项目公示制度和重大项目后评估制度，建立健全建设项目核准与产业准入、土地供应、环保评价相互配套的联动机制，规范政府投资行为。

大力发展非公有制经济。以改善环境、降低门槛为重点，进一步完善鼓励政策，消除体制障碍，努力为非公有制经济发展创造平等竞争的法制环境、政策环境和市场环境。研究制订促进民营经济和中小企业发展的政策措施。鼓励和支持非公有制经济参与公有企业的改制改组，进入金融服务、公用事业、基础设施领域。进一步完善信用担保、人才培训和法律服务等社会服务体系，培育壮大民营企业。年内销售收入过10亿元的民营企业发展到20家以上，民营经济增加值增长18%以上。

（七）继续保持有效投资强度。以促进产业升级、强化薄弱环节、增强发展后劲为目标，进一步加大投资力度，优化投资结构，确保投资规模适度增长、投资效益不断增加。在搞好招商引资的同时，继续加大向上争取力度，利用市国有资产经营公司这一平台，继续加强与国家开发银行等金融部门的合作，推动银企互动，活跃民间资本，多渠道筹措资金。充分发挥财税政策、土地审批和信贷投放的杠杆作用，引导各类资金向高新技术产业和先进制造业倾斜，向农村、农业、生态建设等薄弱环节和基础设施倾斜，向与人民生活密切相关的服务业、社会事业等领域倾斜。围绕以上领域，今年集中力量抓好124个事关全局的重点项目建设。产业领域，重点抓好伽耶造船、威海船厂搬迁等工业项目；交通领域，重点抓好威乳高速、疏港路、荣乌高速公路荣文段、环海生态旅游示范路、威海港三四期、机场改造等项目；能源领域，重点抓好荣成高温气冷堆示范核电站、华能中电和国华风力发电、环翠输变电工程等项目；公益设施领域，重点抓好烟威天然气长输管线及胶东引黄调水工程等项目。同时，做好青烟威城际铁路、中韩铁路轮渡、昆嵛山抽水蓄能电站等项目的前期工作。年内完成规模以上固定资产投资790亿元以上。

（八）大力推进生态市和精品城市建设步伐

加快节约型社会建设。按照减量化、再利用、资源化的原则，广泛开展节能节水节地节材活动，大力发展循环经济和清洁生产，积极推广先进技术和节能设备，促进资源利用方式由线性模式向循环模式转变。年内力争培育出10个循环经济、10个清洁生产试点示范企业。建立“总量控制、定额管理、分类计价、累进加价”的用水制度，年内发展节水农业10万亩，万元生产总值水耗降低8%。深化土地使用制度改革，严格执行投资强度标准，切实做到依法用地、节约用地、集约用地、保障用地。大力倡导绿色消费和节约理念，加大对节能降耗指标的考核力度，推进节约型社会向纵深发展。

加快生态市建设。全面实施《威海生态市建设总体规划》，深入开展“青山、碧海、蓝天、宁静”行动，扎实推进生态市、环境优美镇等系列创建活动，力争今年通过国家生态示范区验收，50%的镇达到省级以上环境优美乡镇标准。搞好米山水库、崮山水库等6大饮用水源地保护和治理，加强对近海水域、滨海湿地、野生动物、古树名木的保护，加大对荣成天鹅湖等自然保护区和森林公园的保护力度。中心市区抓好竹岛河、望岛河等6条主要河道的生态化治理，加强现有污水处理厂管网配套，新建5个小型污水处理厂，搞好医疗废物集中处理工程，完成艾山垃圾处理厂生活垃圾分选及污水综合处理工程。荣成、文登、乳山三个次中心城市也要加强环保设施建设，抓好石岛和俚岛污水处理厂、文登污水处理厂二期和垃圾处理场续建、乳山污水处理厂二期和垃圾渗漏处理等重点工程建设，全面提高城镇污水和垃圾无害化处理能力，年内全市城市污水处理率达到70%。广泛开展全民义务植树活动，继续实施退耕还林、封山育林、荒山绿化、水土流失治理等生态工程，启动乳山7.5万亩荒山绿化工程，抓好威乳高速、成大线等绿色通道建设，搞好环翠楼公园、金线顶公园改造及文化路、海滨路等城市干道绿化上档升级工程，争创“全国绿化模范城市”。全年新增人工造林合格面积16万亩。

加快精品城市建设。按照城市总体规划的要求，搞好各项专项规划和土地利用总体规划的修编工作。强化精品意识，中心市区突出抓好市民文化中心、悦海花园小区、海滨公园改扩建、恢复改造大操场等工程建设，落实城中村改造的政策措施，年内迈出实质性步伐。荣成市抓好公园广场、文体中心续建等工程，文登市抓好新城中心区、河道整治等工程，乳山市抓好文化博览中心、中心绿廊等工程，打造一批人居精品、文化精品和城建精品。要坚持以人为本，中心市区搞好塔山、纪念路等7个居民小区和阮家寺路、西北山路、古陌西路等背街小巷的环境综合整治，在交通量大、过路人流多的地段建设天桥或地下通道。

（九）进一步加强和谐威海建设。按照民主法治、公平正义、诚信友爱、充满活力、安定有序、人与自然和谐相处的要求，建立公平保障人民群众经济、政治和社会利益的机制，努力用亲民的理念和行动来维护人民群众的切身利益，激发全市人民热爱威海、建设威海的热情。

一是千方百计扩大就业。全面落实各项就业再就业政策，进一步完善职业培训和就业服务体系，加快推进市中心人力资源市场及实训基地建设，多渠道开发就业岗位，年内新增就业5万人。严格落实企业最低工资保障和工资指导线制度，建立解决企业拖欠工资问题的长效机制, 切实保障职工的合法利益。

二是健全社会保障体系。扩大基本保险覆盖面，提高基金征缴率和备付能力，年内养老、医疗和工伤保险人数各增加2万人以上。进一步搞好离退休人员社会化管理，确保按时足额领到养老金。健全城乡居民最低生活保障制度，完善失地农民养老保险制度，扩大农村养老保险覆盖面。从今年起，新型农村合作医疗的财政补贴标准由10元提高到40元，相应调整报销比例，增加大病报销额度，增强农民抗大病风险能力。加强困难群众医疗、教育、住房等专项救助。新建改建部分农村敬老院，力争使农村五保集中供养率达到80%以上，愿进全进率达到100%。大力实施光明、助听、助行行动，做好残疾人的健康、就业、奔小康工作。

三是更加重视人口和卫生事业发展。深化医疗卫生体制改革，完善重点疾病预防控制、传染病救治和紧急救援体系。加强县乡医疗卫生机构和乡镇卫生院建设，建立城市双层医疗服务体系，规范医疗服务收费，加强药品价格监管，强化医德医风建设，解决群众看病贵的问题。认真落实农村和城镇失业（无业）人员独生子女父母奖励费财政统一发放政策，抓好农村部分计划生育家庭奖励扶助工作，稳定人口低生育水平，加快推进人口现代化。

四是加快发展文化体育等其他事业。大力弘扬威海精神，积极开展群众性精神文明创建活动，不断提高社会道德水平和文明程度。搞好文化体制改革，大力发展文化产业，继续扶持梦海等艺术精品，打造城市特色文化品牌。积极做好参加全省文艺汇演、省文化产业博览会和21届省运会的准备工作。深入开展全民健身运动，加快推进市体操馆、国家体育总局威海小球类运动项目夏训基地等体育设施建设，吸引更多赛事来威举办。大力推进广播电视和新闻出版工作。依法搞好新一轮地方史志编修工作。继续做好“双拥共建”工作，加强民兵预备役和人民防空建设。关心支持妇女儿童、残疾人和老龄工作。加快发展民族宗教、对台、侨务、档案、气象、防震等各项事业。

五是全面推进“平安威海”建设。坚持重心下移，大力加强社会治安综合治理，深入开展法制宣传教育，积极化解各种矛盾纠纷，切实加强治安防控体系建设，依法严厉打击各种刑事犯罪。加强和谐社区、和谐村镇建设，倡导人与人和睦相处，增强社会和谐基础。继续办好“12345”市长公开电话、市长信箱和行风热线，及时解决群众反映的各种问题。严格落实安全生产责任制，深入开展专项整治，坚决防止重特大安全事故的发生。在去年工作的基础上，进一步健全应急管理体系，整合各类应急资源，切实提高保障公共安全和处置突发事件的能力。

四、切实搞好政府自身建设

站在新起点、实现新发展，对政府工作提出了新的更高要求。各级政府要以贯彻落实《公务员法》为契机，切实加强自身建设，不断提高社会管理和公共服务能力，精心打造顺应时代潮流、体现人民意志的现代政府。

（一）转变职能，依法行政。深入贯彻《行政许可法》，按照政企、政资、政事分开和政府与中介组织分开的原则，加快转变政府职能。要自觉接受人大及其常委会的监督，政协、各民主党派、工商联和人民团体的监督，司法监督和社会监督，认真办理人大议案、建议和政协提案。要依法完善行政管理决策机制，对涉及经济社会发展全局的重大事项，健全协商和协调机制；对专业性、技术性较强的重大事项，健全专家论证、技术咨询、决策评估机制；对与群众利益密切相关的重大事项，健全公示、听证制度。

（二）加强学习，增强本领。所有政府公务员都要勤奋学习，丰富头脑，防止自身知识老化，跟上时代节拍，在建设社会主义新农村、转变经济增长方式、构建和谐威海等诸多方面进行创新。要虚心向基层学习，向群众学习，在丰富的实践中增长才干，增强驾驭全局、处理复杂事务的能力和水平。要深入调查研究，多到困难和问题多的地方去，不断研究新情况，总结新经验，解决新问题，打开新局面。

（三）改进作风，提高效能。要进一步减少和规范行政审批，加强各级行政服务中心建设，切实提高办结时效。要加强和改进科学的绩效考核和评价体系，坚决反对形式主义和虚报浮夸，不提不切实际的目标和口号，不搞劳民伤财的“政绩工程”和“形象工程”。要建立和严格执行行政问责制、责任追究制，重点对政府部门行政首长的决策失误、执行不力、不认真履行职责、内部监督不到位等方面进行问责。要建立完善政府部门的沟通联动机制，加强协调配合，形成干事创业的整体合力。

（四）强化监督，廉洁从政。要围绕涉及群众切身利益的突出问题，认真纠正部门和行业不正之风。要坚持从严治政，严厉查处各类违法违纪案件。今年国家将进行公务员工资制度改革，要坚决执行上级的有关规定，严肃纪律，促进工资制度改革顺利进行。要完善用权、用人、用地、用钱等有关制度，加强对政府投资的重大项目、政府采购以及国有资产重组并购等方面的监督约束，努力从源头上预防和治理腐败，努力建设一个务实高效、清正廉洁、党和人民满意的政府！

各位代表！我们已经迈上“十一五”发展的新征程。让我们在中共威海市委的坚强领导下，高举邓小平理论和“三个代表”重要思想伟大旗帜，以科学发展观统领经济社会发展全局，团结和带领全市人民，开拓创新，锐意进取，真抓实干，奋发有为，为建设一个生机勃勃、充满希望的新威海而努力奋斗！
